# Letter from the Editor in Chief

**DOI:** 10.19102/icrm.2025.16027

**Published:** 2025-02-15

**Authors:** Devi Nair


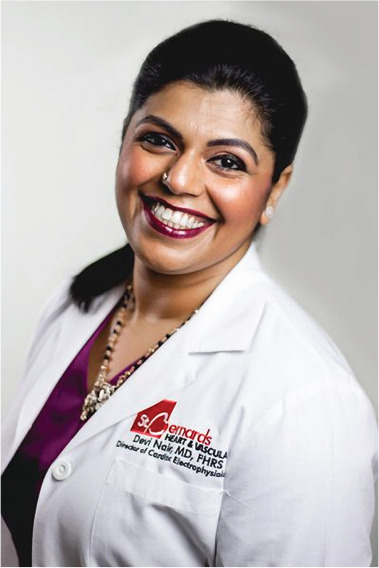


Dear Colleagues,

As we continue into 2025, I am pleased to present the February 2025 issue of *The Journal of Innovations in Cardiac Rhythm Management*, reflecting on the latest advancements in atrial fibrillation (AF) monitoring, management, and procedural innovations. Many of these key discussions were highlighted at the 30th Annual International AF Symposium, which provided a platform for cutting-edge research and clinical insights into pulsed field ablation (PFA), left atrial appendage (LAA) closure, and emerging strategies for AF management.

## Reflections from the AF Symposium: Innovation Meets Clinical Reality

The symposium showcased the continued evolution of AF ablation strategies, emphasizing non-thermal ablation, the integration of stroke risk-reduction strategies, and the optimization of procedural workflows. Some of the key areas of discussion included:

- ***Advancements in PFA.*** PFA remains at the forefront of innovation in non-thermal energy ablation, with ongoing studies evaluating lesion durability, optimal application strategies, and long-term safety. The role of PFA in treating persistent AF and the importance of lesion titration were central topics at the symposium, as researchers seek to define when an ablation set is truly complete.
- ***LAA closure and stroke prevention.*** The integration of LAA closure with AF ablation is becoming an important consideration, with data suggesting the potential benefits of simultaneous procedures in select patient populations. Newer devices and techniques continue to improve procedural efficacy and safety, ensuring more comprehensive stroke-prevention strategies for high-risk AF patients.
- ***Technology-driven AF monitoring and management.*** The rise of wearable technology and artificial intelligence–driven analytics is refining AF detection and long-term rhythm monitoring, allowing for earlier intervention and improved patient engagement.

## The Evolving Landscape of PFA—Innovation with Caution

While PFA is revolutionizing AF ablation, its long-term safety and procedural refinements are still being studied. Several concerns remain, reinforcing the need for continued research and careful clinical application:

- ***Vascular safety.*** Reports of coronary vasospasm following PFA remind us that, while non-thermal energy is designed to minimize collateral damage, it does not eliminate risk entirely. Close procedural monitoring and refinements in energy delivery protocols are necessary to enhance safety.
- ***Hemolysis and renal considerations.*** Data suggest potential hemolysis following PFA, particularly when higher energy levels or prolonged delivery times are used. Understanding its implications for renal function and patient selection will be essential in the long-term adoption of PFA.
- ***Procedural precision.*** Recurrent atrial tachycardias from critical isthmuses created during large-footprint PFA procedures underscore the importance of precise lesion placement and careful titration of energy application to minimize off-target effects.
- ***Combining thermal and pulsed field energies.*** Hybrid ablation approaches that combine radiofrequency (RF) energy with PFA may offer complementary benefits, but the implications of using heterogeneous energy sources within a single procedure remain unclear.
- ***Standardizing lesion titration.*** Unlike RF ablation, PFA lacks standardized markers for determining when a lesion is complete. Developing consistent lesion-titration strategies will be critical for ensuring reproducibility and efficacy across different patient populations.

## This Month’s Featured Articles

In this issue of the journal, we continue to highlight key innovations and challenges in electrophysiology, including:

- ***“Using a Novel Pulsed Field Ablation Technique to Identify the Critical Isthmus in a Tachycardia Circuit.”*** This case report demonstrates how PFA can be leveraged for targeted ablation strategies, minimizing unnecessary lesion delivery.
- ***“Unveiling Rarity: Giant Septal Coronary Vein Dissection, an Unforeseen Twist in Complications of Left Bundle Branch Area Pacing.”*** This cautionary case explores the risks associated with conduction system pacing and how to mitigate potential complications.
- ***“Cardiac Contractility Modulation Therapy and Device Algorithm–related Challenges.”*** This review covers emerging challenges in device therapy for heart failure patients, underscoring the need for broader physician training and troubleshooting frameworks.

## The Road Ahead: Balancing Innovation with Safety

While PFA and other novel AF treatment strategies continue to reshape the landscape of cardiac electrophysiology, we must remain diligent in ensuring patient safety, optimizing procedural workflows, and standardizing best practices. The field is moving rapidly, but careful evaluation, long-term follow-up, and collaborative research efforts are critical to making these technologies truly effective, reproducible, and safe in everyday practice.

I extend my deepest gratitude to all contributors, peer reviewers, and readers who continue to drive innovation and excellence in cardiac rhythm management. I look forward to another year of discovery and collaboration as we refine and advance the tools at our disposal.

Warm regards,


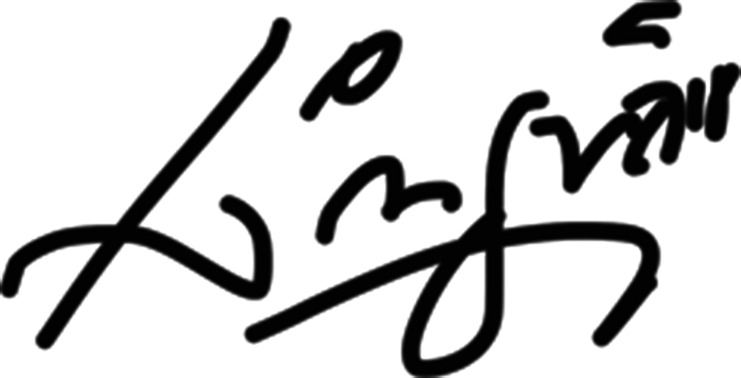


Dr. Devi Nair, md, facc, fhrs

Editor-in-Chief

*The Journal of Innovations in Cardiac Rhythm Management*

Director of the Cardiac Electrophysiology & Research,

St. Bernard’s Heart & Vascular Center, Jonesboro, AR, USA

White River Medical Center, Batesville, AR, USA

President/CEO, Arrhythmia Research Group

Clinical Adjunct Professor, University of Arkansas for Medical Sciences

Governor, Arkansas Chapter of American College of Cardiology

drdgnair@gmail.com

